# Editorial: World Health Day 2023: progress and new perspectives in achieving ‘Health for All'

**DOI:** 10.3389/fpubh.2024.1512006

**Published:** 2024-11-18

**Authors:** María del Carmen Valls Martínez, Martin Ayanore, Hubert Amu, Olatunde Aremu

**Affiliations:** ^1^Mediterranean Research Center on Economics and Sustainable Development (CIMEDES), Economics and Business Department, University of Almería, Almería, Spain; ^2^Fred N. Binka School of Public Health, University of Health and Allied Sciences, Hohoe, Ghana; ^3^Department of Population and Behavioural Sciences, Fred N. Binka School of Public Health, University of Health and Allied Sciences, Hohoe, Ghana; ^4^Department of Public Health, Faculty of Health Education and Life Sciences, Birmingham City University, Birmingham, United Kingdom

**Keywords:** health and wellbeing, public health, Health for All, health service utilization, WHO, universal coverage, life expectancy, income inequalities

As we celebrate World Health Day 2023, the “Health for All” theme underscores the global commitment to ensuring that every individual has access to essential health services regardless of background or socioeconomic status. World Health Day 2023 coincides with 75 years when the World Health Organization (WHO) was established as the first United Nations specialized agency, with the constitution becoming enforceable on 7th April 1948. WHO's constitution asserts that the highest attainable standard of health is a fundamental right of every human being (1). Although considerable progress has been made in advancing public health worldwide, the journey toward universal health coverage (UHC) is far from complete (2–4). The central tenet of “Health for All” envisions that people everywhere have good health for a fulfilling life in a peaceful, prosperous, and sustainable world while recognizing and promoting the right to good health is necessary (5). More than a third of the world's population is currently lacking essential health provisions, with no fewer than 2 billion people in precarious situations of having no healthcare coverage safety net such as health insurance coverage (2, 6, 7). As the world advances toward achieving “Health For All,” it is crucial to reflect on the progress achieved, as well as how the world can continue to guide and support this goal by addressing existential health challenges such as recent pandemic of COVID-19—and any future health emergencies (8).

In this spirit, Frontiers in Public Health launched this article Research Topic to coincide with this UN day, highlighting progress and new perspectives to accelerate progress in achieving “Health for All,” with a particular interest in the world's most vulnerable populations. In this Research Topic of *Frontiers in Public Health*, which attracted 10 articles, we highlight the progress made, the challenges that persist, and the innovative approaches shaping the future of global health.

## Addressing health inequalities for vulnerable populations

A key theme in this Research Topic is the persistent health inequalities faced by vulnerable populations. In the article “*God is my only health insurance”: a mixed-methods study on the experiences of persons with disability in accessing sexual and reproductive health services in Ghana*, the authors explored the significant barriers people with disabilities (PwDs) encounter in accessing sexual and reproductive health (SRH) services. The study found that only 33.8% of PwDs in Ghana's Ashanti Region used SRH services, with stigma, discrimination, and inadequate healthcare infrastructure being significant obstacles. The article calls for reforms to make healthcare more inclusive by training healthcare providers, improving the accessibility of facilities, and enhancing national health insurance schemes (Seidu et al.).

Another study, *Work-related challenges and their associated coping mechanisms among female head porters (Kayayei) in Ghana* investigates the health challenges faced by female porters and internal migrants in Ghana. The study highlights the physical and mental health issues arising from poor working conditions and limited access to healthcare. The authors recommend collaborations between government and non-governmental organizations to raise awareness, provide social support, and promote safer working conditions for these women (Komesuor et al.).

## The role of public health services in marginalized communities

The importance of robust public health systems in improving the wellbeing of marginalized populations is another central focus. In the study, *Does improving basic public health services promote household consumption of rural migrant workers? Evidence from China*, the authors examined how access to Basic Public Health Services (BPHS) can improve the living conditions of rural migrant workers by boosting household consumption. The study underscores the dual benefits of public health services: they improve health outcomes and contribute to economic empowerment, particularly for socially vulnerable groups (Pan et al.).

Similarly, the article *Community-based Health Planning and Services programme in Ghana: a systematic review* evaluates Ghana's Community-based Health Planning and Services (CHPS) program, which is the main vehicle through which primary healthcare is provided at the community level. The systematic review concludes that CHPS has been successful in overcoming barriers to healthcare access, such as distance and healthcare worker shortages, and serves as a model for other countries aiming to strengthen primary healthcare in rural areas (Adusei et al.).

## The digital economy and public health

The rapid advancement of the digital economy presents both opportunities and challenges for public health. In the article “*The impact of digital economy development on public health: evidence from Chinese cities*,” the authors explore how digital economic growth can positively influence public health outcomes. By improving access to health information and promoting innovation, the digital economy has the potential to enhance healthcare delivery. However, the study also highlights the need to bridge the digital divide to ensure that rural and underdeveloped regions benefit from these advancements (Li and Li).

Another article, “*Benchmarking medical laboratory performance on a global scale,”* delves into the need for standardization and digitalisation in medical laboratories worldwide. The study reveals that many laboratories lack key performance indicators (KPIs) to monitor diagnostic speed and accuracy, emphasizing the importance of adopting digital tools and automation to improve patient outcomes and operational efficiency. Overall, the study posits that, the benchmark elucidates current practice and has the potential to guide improvement efforts and standardization in quality and safety for patients and employees alike, as well as the sustainability of healthcare systems around the globe (Huf et al.).

## Healthcare reforms and reducing health inequality

Several articles focus on the role of healthcare reforms in reducing health inequality. In the article “*Public hospital reform, family health consumption and health inequality: evidence from China Family Panel Studies,”* the authors examined how China's 2017 public hospital reforms, aimed at reducing medical costs, have helped to alleviate health inequality. The study shows that these reforms have reduced household medical expenses and encouraged healthier lifestyles by increasing spending on health-related leisure activities. Thus, the authors conclude that reform policies in the medical field should integrate with policies promoting basic education, developing the silver economy, and advancing the health industry to work synergistically for better outcomes for patients (Jiang et al.)

Similarly, the article “*Impact of Urban-Rural Resident Basic Medical Insurance on Consumption Quality of Middle-aged and Older Adult Residents: Evidence from Rural China”* evaluates the effects of the Urban-Rural Resident Basic Medical Insurance (URRBMI) scheme. The findings indicate that URRBMI has significantly enhanced healthcare access for rural, middle-aged, and older adult residents, particularly for non-food and health-related consumption. These reforms are crucial for reducing the health disparities between urban and rural populations and among different income groups (Zhou and Ping).

## Innovations in public health screening

Innovation in public health screening is another critical area of focus. In the article “*Cost-effectiveness of portable-automated ABR for universal neonatal hearing screening in India*,” the authors assess the effectiveness of using portable auditory brainstem response (P-AABR) devices for neonatal hearing screening. The study concludes that P-AABR is both cost-effective and efficient, particularly in remote areas with limited healthcare infrastructure. Implementing such technologies could greatly enhance early detection of hearing impairments and reduce long-term healthcare costs (Sahoo et al.).

## Education, income inequality, and life expectancy: insights from the EU

The article “*Impact of education and Income Inequalities on life expectancy: insights from the new EU members”* examines the relationship between social inequalities and population health in the context of the European Union's newer member states. The study identifies that higher levels of education and income inequality negatively impact life expectancy. In contrast, factors such as internet usage and mobile phone penetration have a positive effect, underscoring the critical role of information and communication technology (ICT) in improving public health. The findings suggest that reducing inequalities in education and income while promoting greater access to digital resources could be critical strategies in extending life expectancy and improving population health (Sart et al.).

## Conclusion: moving toward universal health coverage

We, the Editors, believe that the range of varied Research Topics contributed by different authors from different parts of the world featured in this Frontiers in Public Health issue presents a nuanced picture of the global efforts to UHC (9). Ultimately, this may open a new vista of knowledge to understanding the ever-increasing need to ensure all individuals have access to the healthcare needed for optimum health and better quality of life. There is no doubt that significant barriers remain while substantial progress has been made, particularly in expanding public health services and leveraging digital technologies (6, 10). Advancements in the deployment of digital technologies have ushered in new hope for ensuring people can access healthcare without geographical boundaries. Despite all these, inequities persist along the lines of income, education, geography, and gender, disproportionately affecting vulnerable populations (6, 10, 11). Health policymakers, practitioners, and communities must work together to address these challenges. The insights provided in these studies emphasize the need for targeted interventions that address the specific barriers faced by marginalized populations, along with broader systemic reforms. Investing in innovative healthcare delivery systems, promoting public health education, and fostering cross-sector collaboration can make meaningful strides toward realizing the “Health for All” goal and ensure that everyone benefits from more equitable healthcare and good health.
